# Supplementary material for: A Small Molecule, 4-Phenylbutyric Acid, Suppresses HCV Replication via Epigenetically Induced Hepatic Hepcidin
Source: Int J Mol Sci. 2020 Aug 1;21(15):5516. doi: 10.3390/ijms21155516 (PMC7432483; doi:10.3390/ijms21155516)
Supplement: Supplementary file 1 [file ijms-21-05516-s001.pdf]

Supplementary Materials and Methods

Determination of Cell Viability

Cell viability was assessed by lactate dehydrogenase (LDH)-release assays. LDH-release assays (Promega) were performed under the same conditions according to manufacturers' instructions. Each value represents the mean of a minimum of nine wells.

Immunoprecipitation

hVAP-33, NS5A or NS5B co transfected cells were incubated with 4-PBA for 24h. Treated cells were lysed with lysis buffer, and cell lysates were cleared by centrifugation at 14,000 rpm for 15 min. The lysates (2 mg) were incubated with anti-NS5A or -NS5B antibody (10 μg) for 12 h at 4°C. Protein-A/G-conjugated agarose beads were added, followed by incubation for 5h at 4°C. Beads were washed three times with 1× TBST. Immunopellets were boiled with SDS-PAGE sample buffer and resolved by electrophoresis.

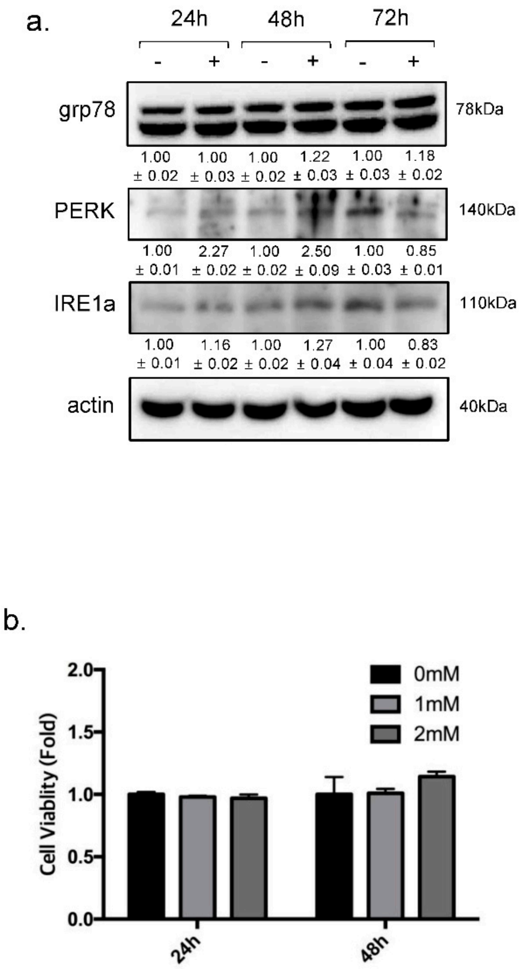

Figure S1. a) Huh7.5-Con1 cells were treated for indicated times with 1 mM 4-PBA. ER stress marker (grp78, PERK and IRE1a) expression was evaluated by immunoblotting; actin was used as a loading control. b) Huh7.5-Con1 cells were treated with 0, 1, or 2 mM 4-PBA for 24 or 48 h, and cell viability was evaluated with the LDH release assay. Data are expressed as mean ± SD of five independent experiments.

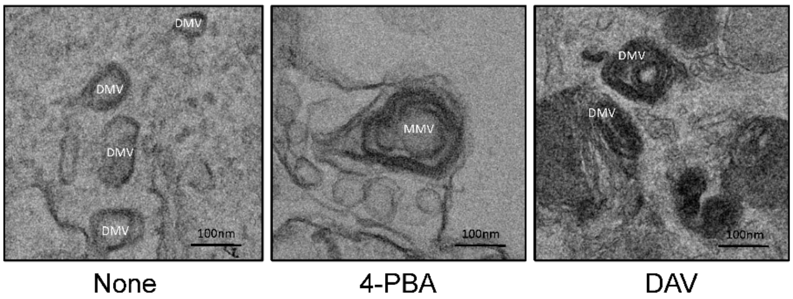

Figure S2. Huh7.5-Con1 cells without (left) or with 1 mM 4-PBA (middle) or 2 μM DAV (right) treatment for 24 h. DMVs were detected by electron microscopy. DMV, double membrane vesicle; MMV, multi-membrane vesicle.

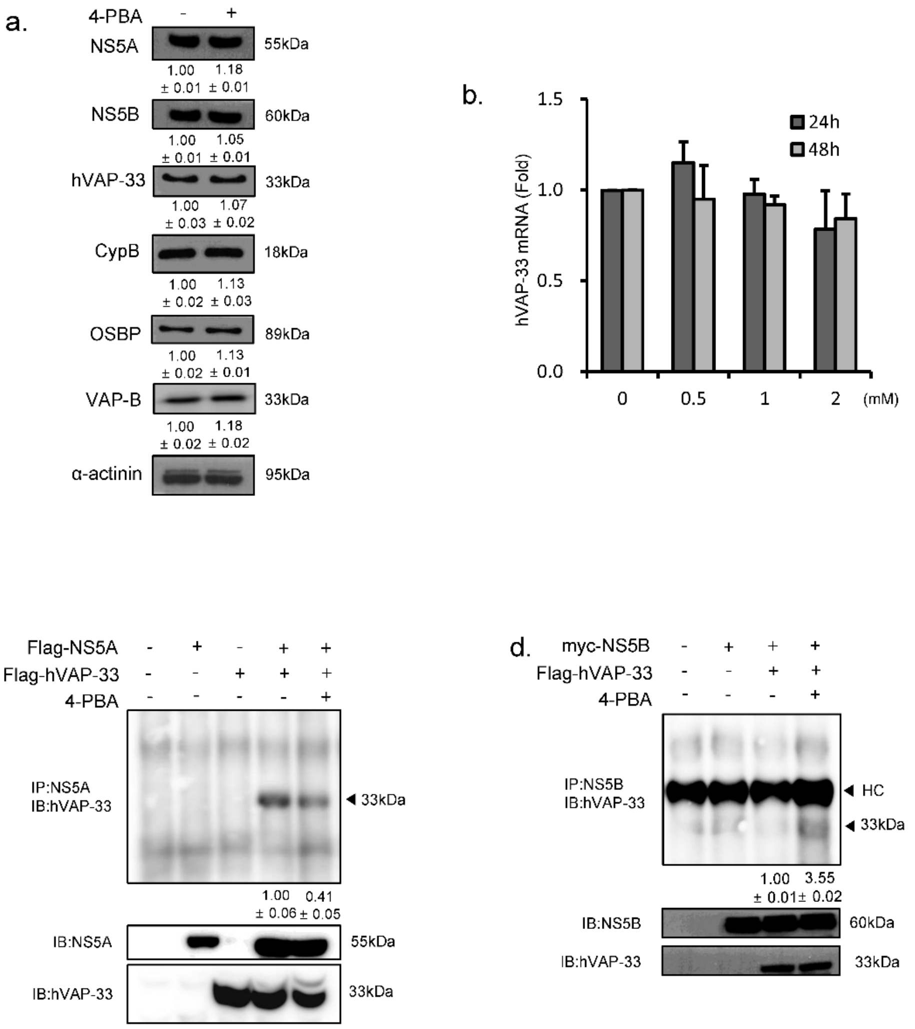

Figure S3. a) Huh7.5-Con1 cells were treated with 4-PBA for 24 h. Expression of NS5A, NS5B, hVAP-33, CypB, OSBP and VAP-B was evaluated by immunoblotting 5% of each immunoprecipitated input. b) Huh7.5-Con1 cells were treated with up to 2 mM 4-PBA for 24 or 48 h. hVAP-33 mRNA level was measured by qRT-PCR. Data are expressed as mean ± SD of three independent experiments. c, d) HEK293T cells were transfected with Flag-NS5A, Flag-hVAP-33, or both. Immunoprecipitation was carried with transfected cells with or without 4-PBA treatment for 24 h. Protein complexes immunoprecipitated with antibodies against NS5A (c) or NS5B (d) were probed with anti-hVAP-33 antibody.

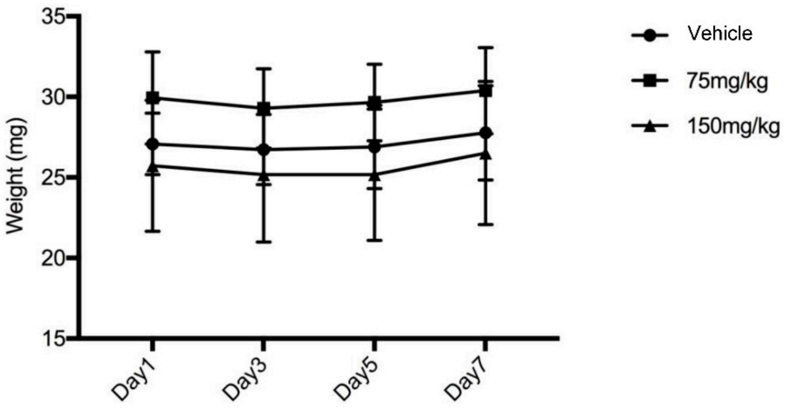

Figure S4 Body weight of HCV infection model mice was measured 1, 3, 5, and 7 days after 4-PBA treatment.
